# Comparison of Patient and Surgeon Expectations of Total Hip Arthroplasty

**DOI:** 10.1371/journal.pone.0030195

**Published:** 2012-01-17

**Authors:** Claire Jourdan, Serge Poiraudeau, Stéphane Descamps, Rémy Nizard, Moussa Hamadouche, Philippe Anract, Stéphane Boisgard, Myriam Galvin, Philippe Ravaud

**Affiliations:** 1 AP-HP, Hôpital R. Poincaré, Service de Médecine Physique et Réadaptation, Garches, France; 2 CHU Clermont-Ferrand, Hôpital Gabriel Montpied, Service d'Orthopédie, Traumatologie, Chirurgie plastique et reconstructive, Université Claude Monnet, Clermont-Ferrand, France; 3 AP-HP, Hôpital Lariboisière, Service de Chirurgie Orthopédique et Traumatologique, Université Paris 7, Paris, France; 4 AP-HP, Hôpital Cochin, Service d'Orthopédie, Université Paris Descartes, Paris, France; 5 Institut National de la Santé et de la Recherche Médicale (INSERM) E 0738, Centre d'Epidémiologie Clinique, Université Paris Descartes, Paris, France; University of Michigan, United States of America

## Abstract

**Objectives:**

Analysis of discrepancies between patient and surgeon expectations before total hip arthroplasty (THA) should enable a better understanding of motives of dissatisfaction about surgery, but this question has been seldom studied. Our objectives were to compare surgeons' and patients' expectations before THA, and to study factors which affected surgeon-patient agreement.

**Methods:**

132 adults (mean age 62.8+/−13.7 years, 52% men) on waiting list for THA in three tertiary care centres and their 16 surgeons were interviewed to assess their expectations using the Hospital for Special Surgery Total Hip Replacement Expectations Survey (range 0–100). Patients' and surgeons' answers were compared, for the total score and for the score of each item. Univariate analyses tested the effect of patients' characteristics on surgeons' and patients' expectations separately, and on surgeon-patient differences.

**Results:**

Surgeon and patient expectations' mean scores were high (respectively 90.9+/−11.1 and 90.0+/−11.6 over 100). Surgeons' and patients' expectations showed no systematic difference, but there was little agreement on Bland and Altman graph and correlation coefficient was low. Patients had higher expectations than surgeons for sports. Patients rated their expectations according to trust in physician and mental quality of life, surgeons considered disability. More disabled patients and patients from a low-income professional category were often “more optimistic” than their surgeons.

**Conclusion:**

Surgeons and patients often do not agree on what to expect from THA. More disabled patients expect better outcomes than their surgeons.

## Introduction

Total hip arthroplasty (THA) is the most effective treatment for disabling hip osteoarthritis [1]. Main goals of THA are to reduce pain and increase patients' functional abilities. Patient's preferences play a pivotal role in surgery decision making, and the assessment of patients expectations regarding the functional benefits of THA is recommended [2].

Expectations in this domain can be understood as “expectancies” (or “probability expectations”), meaning patients' judgments about the benefits of surgery, or “value expectations”, meaning patients' desires, hopes, or wishes concerning outcome. This last approach is close to “importance”, which refers to patients' priorities concerning function post-surgery [3].

Patient's expectations about THA have been shown to depend on several demographic, clinical, psychological and socioeconomic factors [4]–[6]. Patients ethnical origins, employment status, and trust in surgeon have a significant effect on expectations about THA [5]. Patients with worse disease severity have been shown to have higher expectations scores than those with less severe disease [6]. Expectations can also vary from one country to another, as shown in total knee arthroplasty [7].

The relationship between pre-operative expectations and later satisfaction with surgery is complex. Optimistic expectations can be an independent predictor of better joint arthroplasty outcome [4], [8]. However, satisfaction with surgery is also associated with the proportion of fulfilled expectation [9], and unmet expectations can cause dissatisfaction [10], [11].

Moreover, patients with low expectations might decline surgery although they would benefit from it, as expectations determine willingness to undergo surgery [12]–[14]. It is therefore important to determine which patients have high or low expectations about THA compared to their surgeons, in order to maximize THA indication and outcomes. One way to address this question is to better understand the differences between patients' and surgeons' views on the expected results of THA.

While it is established that patients suffering from osteoarthritis and their physicians might differ in their assessment of important health and symptom status [15], and of surgical outcome [16], [17], the difference between patients' and surgeons' expectations before THA has seldom been studied. Moran and colleagues [18], found that surgeons had better expectations of predicted postoperative functional scores than patients. Street and colleagues [19] showed that patient-surgeon concordance regarding the expected benefits of surgery was poor, and differed with quality of communication, especially surgeon's information giving. These studies used different means of evaluating patient's expectations: functional “expected” scale or close-ended questions. Assessments of expectations have used various methods until recently, when patient-derived scales have been validated for patients' “probability” expectations [6], [20] or for patients' concerns and “value” expectations [21].

This study aimed to compare surgeons' and patients' “probability expectations” of THA using a validated patient-derived questionnaire. Its first objective was to assess how much, in which direction, and for which items these could differ. Its second objective was to study which parameters explained patient-surgeon differences in expectations.

## Methods

This cross-sectional survey was approved by the ethical committee of the Institutional Review Board of APHP Bichat Hospital, Paris. Participants gave oral informed consent before telephone interview, followed by written informed consent through post.

### Population

Patients and surgeons were recruited from three French tertiary care orthopaedic centres (APHP Hospital Lariboisière, Paris, APHP Hospital Cochin, Paris, and Hospital Gabriel Montpied, Clermont-Ferrand) between January and July 2009. Surgeons participating in the study included consecutive adult patients on waiting list for hip replacement surgery. Exclusion criteria were tumoral, infectious or inflammatory disease of the hip, revision THA surgery, and patient's refusal or inability to answer the questionnaire.

### Evaluation

Expected benefits of hip surgery were assessed using the Hospital for Special Surgery Total Hip Replacement Expectations Survey (THR Survey) [6], [20], adapted to French by back translation [22]. In this patient-derived questionnaire, patients are asked the following question: “How much relief or improvement do you expect in the following areas as a result of hip replacement surgery?” The scale contains 18 items addressing symptoms, daily function, exercise, employment, and psychological well-being, and the answers range from “patient does not have this expectation, or this expectation does not apply” (scoring 0) to “complete improvement or back to normal” (scoring 4). We chose to separate the answer “patient does not have this expectation” (scoring 0) from the answer “this expectation does not apply” (“not applicable item”).

The same questionnaire was used by the patient's surgeon, with a question modified as follows: “How much relief or improvement seems realistic to you in the following areas as a result of hip replacement surgery for this specific patient?” The 18 items and their answers were identical.

Patients' and surgeons' expectation scores were calculated by summing the scores of all the applicable items, higher scores indicating higher expectations. Scores were transformed by the formula: (sum/number of applicable items for the patient×4)×100, to obtain scores ranging from 0 to 100, as described before [23]. In order to compare patients' and surgeons' scores, the items considered “not applicable” by a patient were considered “not applicable” in the surgeon's assessment also. Differences in expectations were defined as: surgeon's score - patient's score (a positive difference suggested that the surgeon had higher expectations than his patient and *vice versa*).

Demographic characteristics included gender, retirement status, professional category (liberal or senior officer versus employee or worker), marital status (married or in couple versus single), educational level (pre-secondary level versus post-secondary level), and the physical activity that the patient wished to resume after surgery (recommended or not after hip arthroplasty, according to The Hip Society and the American Association of Hip and Knee Surgeons [24]).

Health status evaluation included age, Body Mass Index (inferred from patients' reports of height and weight), and average hip pain during the last four weeks on a numeric rating scale (0–10). Co-morbidities were measured using the Charlson Co-morbidity Index [25], dichotomized as zero (no relevant co-morbidity) versus one or more co-morbidities. A history of ipsilateral hip arthroplasty was recorded.

Functional evaluation used the short 8-item Western Ontario and Mac Master Universities (Womac) function subscale [26], [27], which ranges from 0 (no disability) to 32 (extreme disability). Quality of life was assessed by the Medical Outcome Study Short Form-12 (SF-12) [28], [29], for which higher scores indicate better quality of life.

To measure patient-surgeon communication during the clinical visit, patient self-report measures were used, with previously described questions [19]: five questions measured patient's perception of their own involvement during visit, three questions measured patient's perception of the surgeon's partnership building, and five questions measured patient's perception of information given by the surgeon. Each question allowed three answers: “disagree”, “neither agree nor disagree”, “agree”. Scores (1–3) were summed for each dimension of communication. Patients' trust in their surgeon was measured by a numeric rating scale (0–10).

Patients were recruited consecutively after their clinical visit with their surgeons and before surgery. Visits did not differ from usual, no specific questions were asked. They were interviewed by phone by a unique independent assessor, using a standardized questionnaire, to assess their expectations of surgery, and socio-demographic and health status characteristics. The attending surgeon for each patient was asked to fill in the THR Survey, to assess the benefit which their patient could reasonably expect. Surgeons' completed the survey a few days after clinical visit, using their own clinical record. The patient and the assessor were unaware of the surgeon's responses and *vice versa*. Clinical reports were reviewed to collect medical data.

### Statistical analysis

Description of the sample characteristics and expectation scores used mean and standard deviation (SD) for continuous variables, and counts and percentages for categorical variables.

Considering that expectations of results of THA had been evaluated by two judges, the patient and his surgeon, concordance between surgeons' and patients' total expectations was judged using an intra-class correlation coefficient and the Bland and Altman method [30], and correlation between surgeons' and patients' expectations was assessed by the Pearson correlation coefficient. An aggregated score of surgeon-patient discrepancies was computed for each patient, using the sum of the absolute values of surgeon-patient differences for each item, corrected for the number of applicable items.

Differences (mean, 95% confidence interval (CI)) between surgeon's and patient's rating of expectations for each separate item were described.

We studied which parameters, among the variables previously stated, were associated with either patient expectations' scores or with surgeon expectations' scores separately. Spearman correlation coefficients were used for continuous variables, and ANOVAs (or Kruskal Wallis tests if necessary) for categorical variables. Alpha error limit was set at 5%.

To study surgeon-patient discrepancies in expectations, we used surgeon-patient difference in total expectation score, as defined above. We described patients according to the tertiles of this difference, thus dividing the population into three groups of equal size (n = 44). The group of patients with lowest (negative) surgeon-patient differences was classified as “more optimistic”, the group with highest differences was classified as “more pessimistic”, while the middle group had low surgeon/patient discrepancies. We compared the two extreme groups of patients (“optimistic” vs. “pessimistic”) using chi square tests for categorical variables, and ANOVAs for continuous variables.

Surgeons' effect on differences in expectation was described using a box plot. To account for surgeons' effect, the same variables were tested through mixed effect logistic regression models, with “optimistic” vs. “pessimistic” as the dependant variable, the patient being analysed as nested within his surgeon (fixed effect were patients characteristics, random effects were surgeons).

As missing data were scarce (0 to 4%), analyses were realized on complete data. The statistical software SAS® version 9.1 (SAS Institute, Cary, North Carolina) and R® version 2.8.1 were used.

## Results

### Population characteristics

A total of 16 surgeons agreed to participate and screened 202 patients. Seventy were not included: 22 patients were undergoing revision surgery, 6 had inflammatory or infectious disease of the hip, 9 patients declined to participate, 5 were unable to answer, 26 were impossible to contact before the surgery, and 2 had their surgery cancelled before the interview. The final sample consisted of 132 patients: respectively 63 (48%), 50 (38%) and 19 (14%) from each centre.

Mean +/− SD age was 62.8±13.7 years, ages ranging from 19 to 87 years. Patients were male in 52%. Indications for surgery were mostly primary or secondary hip osteoarthritis (82%) and avascular necrosis (12%). Patients were interviewed on average 37 days before surgery. Patients' characteristics and survey scores are shown in table 1. Patients' characteristics did not significantly differ according to centres or surgeons (data not shown).

**Table 1:** Characteristics of study population (n = 132).*

Socio-demographic characteristics	Number (%)
History of ipsilateral THA	19 (14.4%)
Charlson Comorbidity Index : score = 0	72 (54.5%)
Retired	85 (64.4%)
Profession : liberal or senior officer	75 (56.8%)
Education : post secondary level	63 (47.7%)
Married or in couple	82 (62.1%)
Sport : not recommended after THA	16 (12.1%)

Health status and survey scores		Mean ± standard deviation
Body Mass Index (kg/m^2^)		25.6±4.1
Trust (NS, max = 10)		9.2±1.0
Communication	Patient participation (max = 15)	8.0±2.7
	Partnership building (max = 9)	5.6±2.1
	Information (max = 15)	11.1±3.0
Pain (NS, max = 10)		6.8±1.9
WOMAC (max = 32)		18.7±5.4
SF-12 : PCS (max = 100)		32.2±7.9
SF-12 : MCS (max = 100)		48.6±11.4

*THA = Total Hip Arthroplasty; NS = Numeric Scale; WOMAC = Western Ontario and McMaster Universities Osteoarthritis Index; SF-12 = Medical Outcome Study Short Form-12; PCS = Physical Component Summary; MCS = Medical Component Summary.

### Patients' and surgeons' expectations

**Figure 1:**
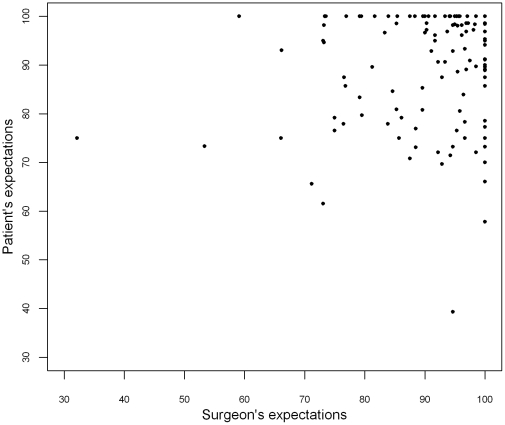
Scatter plot of patients' versus surgeons' expectations.

**Figure 2:**
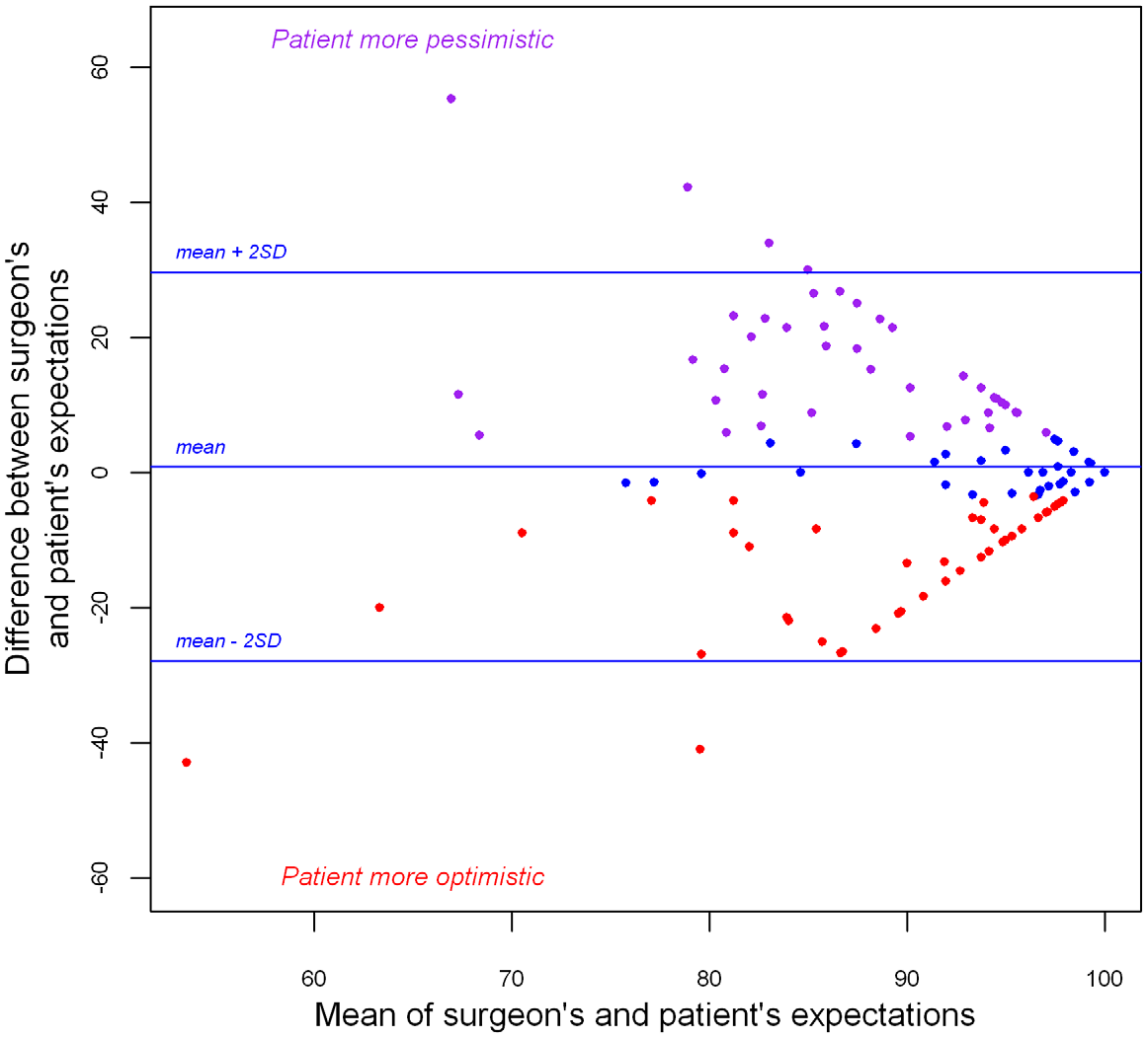
Bland and Altman graph of differences in expectations (surgeon's score – patient's score). For each patient, X-axis represents mean (½×(surgeon's score+patient's score)), Y-axis represents difference. The three tertiles-defined groups of patients are represented by three colors (red = “more optimistic patients”; blue = middle group, purple = “more pessimistic patients”).

Surgeons' and patients' expectations scores were both high, respectively 90.9±11.1 and 90.0±11.6. No systematic bias, but marked discrepancies between surgeons' and patient's expectations in both directions were found (figures 1 and 2), and mean aggregated score of surgeon-patient discrepancy was 9.3±7.3 (range = [0–39.9]). Intra-class correlation coefficient was low (0.16; CI_95%_ = [−0.03; 0.33]), and Pearson's coefficient revealed no significant correlation (rho = 0.17, p = 0.06). On the Bland & Altman graph (figure 2), the differences in expectations (surgeon's score – patient's score) were distributed around zero and ranged from −42.9 to 55.4. Greatest absolute value of surgeon-patient differences were observed for lowest mean expectation scores, while patients with high expectations showed less surgeon-patient differences.

**Figure 3:**
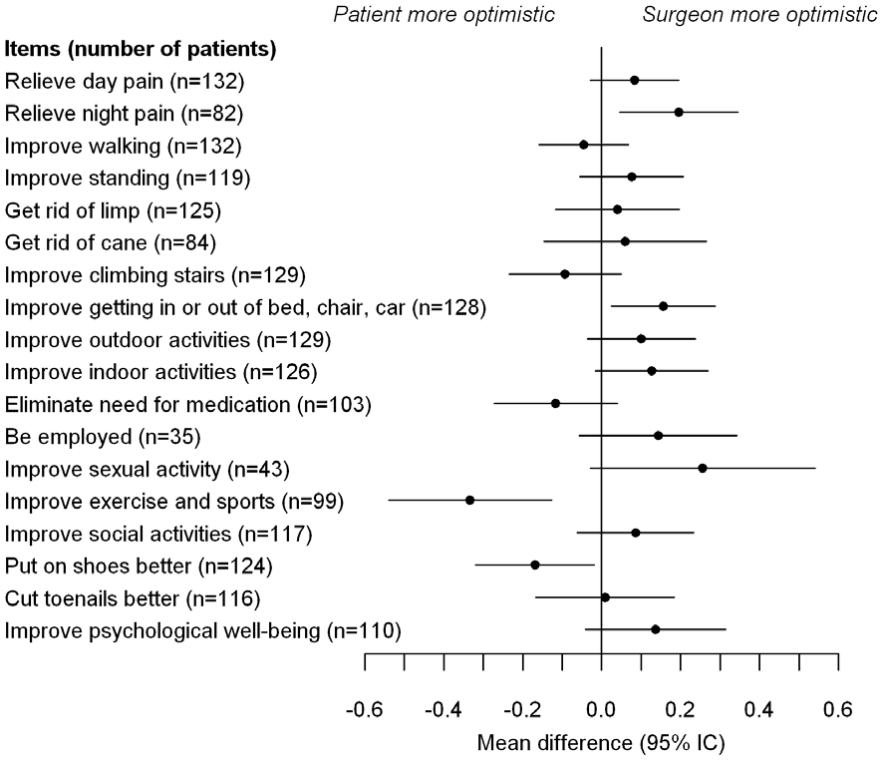
Mean (95% CI) surgeon – patient differences for each item of the THR Survey. Scores for each item range from 0 to 4. N = number of patients who stated this item as “applicable”.

Surgeon-patient differences for each item are shown in figure 3. Greatest divergences were found for item 14 (“Improve ability to exercise or participate in sports”), patients being there more optimistic than surgeons.

### Factors affecting patient's or surgeons' expectations separately (table 2)

**Table 2:** Determinants of patients' or surgeons' expectation (total THR Survey score, max = 100).*

Socio-demographic characteristics		Patients' expectations		Surgeons' expectations	
		mean ± standard error	*p*	mean ± standard error	*p*
Recrutement site	Cochin	89.9±1.4	ns	**94.5±0.9**	**<0.01**
	Lariboisière	89.8±2.8		**86.5±1.8**	
	Gabriel Montpied	91.0±2.4		**90.4±2.8**	
Gender	Male	88.5±1.5	ns	91.2±1.5	ns
	Female	91.6±1.3		90.5±1.2	
Ipsilateral THA	Yes	90.5±2.4	ns	89.8±2.3	ns
	No	90.0±1.1		91.1±1.1	
Comorbidities	Score = 0	**92.4±1.2**	**<0.01**	92.1±1.1	ns
	Score > 0	**87.0±1.7**		89.3±1.7	
Retirement	Yes	89.6±1.3	ns	92.2±1.5	ns
	No	90.7±1.6		90.2±1.3	
Profession	Liberal, senior officer	89.9±1.2	ns	**92.5±1.3**	**<0.01**
	Employee, worker	90.1±1.7		**88.7±1.5**	
Education	Post secondary	89.3±1.4	ns	91.0±1.5	ns
	Pre secondary	90.7±1.4		90.8±1.3	
Marital status	Married/in couple	89.9±1.4	ns	**92.4±1.1**	**<0.05**
	Single	90.3±1.5		**88.4±1.8**	
Sport	Recommended	90.0±1.1	ns	94.6±3.0	ns
	Not recommended	90.4±2.4		98.4±1.0	

Health status and survey scores		Patients' expectations		Surgeons' expectations	
		Spearman correlation	*p*	Spearman correlation	*p*
Age		**−0.17**	**<0.05**	**−0.18**	**<0.05**
Body Mass Index (kg/m^2^)		−0.07	ns	0.04	ns
Trust in surgeon (NS, max = 10)		**0.19**	**<0.05**	−0.08	ns
Communication	Patient participation	0.07	ns	−0.10	ns
	Partnership building	0.06	ns	−0.02	ns
	Information	0.06	ns	−0.15	ns
Pain (NS, max = 10)		0.12	ns	−0.06	ns
WOMAC (max = 32)		0.13	ns	**−0.20**	**<0.05**
SF-12 : PCS (max = 100)		−0.10	ns	**0.25**	**<0.01**
SF-12 : MCS (max = 100)		**0.21**	**<0.05**	0.01	ns

*Bold characters indicate significative tests; NS = Numeric Scale; WOMAC = Western Ontario and McMaster Universities Osteoarthritis Index; SF-12 = Medical Outcome Study Short Form-12; PCS = Physical Component Summary; MCS = Medical Component Summary.

Patients' and surgeons' scores in expectations were both significantly associated with age (both p value<0.05), expectations being lower for older patients. Trust in surgeon (p<0.05), SF-12's mental component (p<0.05), and comorbidity index (p<0.01) significantly influenced patients' expectations. Surgeons' scores were associated with Womac (p<0.05), SF-12's physical component (p<0.01), marital and professional status (p<0.05 and <0.01, respectively). Surgeons' expectations were lower concerning more disabled patients (p<0.05).

**Figure 4:**
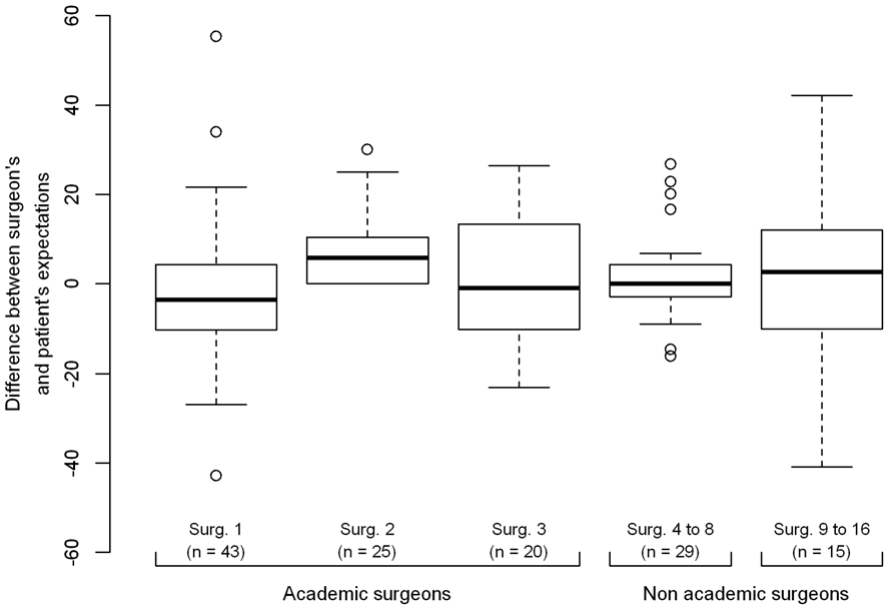
Box plots comparing surgeon-patient differences between surgeons. Boxes include the first quartile, the median and the third quartile. Surgeons 4 to 8, who recruited 5–11 patients each, and surgeons 9 to 16, who recruited 1–3 patients each, were aggregated.

Surgeons' expectations were significantly different amongst different recruitment sites (p<0.01) or amongst surgeons themselves (p<0.001 for Kruskal-Wallis test). Patients' expectations did not show any significant difference between surgeons or sites. Surgeons' effect was also significant on surgeon-patient differences in expectations (see figure 4, p = 0.003 for Kruskal-Wallis test).

### Factors affecting surgeon-patient agreement: comparison between patients markedly more optimistic and patients markedly more pessimistic (table 3)

**Table 3:** Comparison between patients more optimistic than his surgeon and patients more pessimistic.

Patients characteristics		More optimisticpatients (n = 44)	More pessimisticpatients (n = 44)	p-value(univariate tests)^1^	p-value(adjusted for surgeon effect)^2^
**Socio-demographic characteristics**					
Recrutement site	Cochin	**12 (31%)**	**27 (69%)**	**0.003**	**-**
	Lariboisière	**27 (69%)**	**12 (31%)**		
	Gabriel Montpied	**5 (50%)**	**5 (50%)**		
Gender	Male	19 (43%)	25 (57%)	0.3	0.2
	Female	25 (57%)	19 (43%)		
Ipsilateral THA	Yes	7 (47%)	8 (53%)	1	0.6
	No	37 (51%)	36 (49%)		
Comorbidity Index	Score = 0	24 (59%)	17 (42%)	0.2	0.07
	Score>0	19 (41%)	27 (59%)		
Retirement	Yes	33 (53%)	29 (47%)	0.5	0.2
	No	11 (42%)	15 (58%)		
Profession	Liberal, senior officer	**19 (38%)**	**31 (62%)**	**0.02**	**0.03**
	Employee, worker	**25 (66%)**	**13 (34%)**		
Education	Post secondary	18 (44%)	23 (56%)	0.4	0.4
	Pre secondary	26 (55%)	21 (45%)		
Marital status	Married/in couple	22 (45%)	27 (55%)	0.4	0.2
	Single	22 (56%)	17 (44%)		
Sport	Recommended	41 (52%)	38 (48%)	0.5	0.1
	Not recommended	3 (33%)	6 (67%)		
**Health status and survey scores**					
Age		65.3±2.2	64.4±2.0	0.8	0.4
Body Mass Index (kg/m^2^)		25.2±0.8	25.9±0.5	0.4	0.9
Trust in surgeon (NS, max = 10)		9.4±0.1	9.1±0.2	0.2	0.1
Communication	Patient participation	8.3±0.5	7.6±0.4	0.3	0.7
	Partnership building	5.7±0.3	5.7±0.3	0.9	0.8
	Information	11.7±0.5	10.8±0.5	0.2	0.8
Pain (NS, max = 10)		7.0±0.3	6.4±0.3	0.2	0.1
WOMAC (max = 32)		**20.4±0.8**	**17.2±0.8**	**0.006**	**0.006**
SF-12 : PCS (max = 100)		**29.4±1.1**	**34.6±1.3**	**0.003**	**0.005**
SF-12 : MCS (max = 100)		50.7±1.7	47.8±1.8	0.3	0.4

Bold characters indicate significative tests; NS = Numeric Scale; WOMAC = Western Ontario and McMaster Universities Osteoarthritis Index; SF-12 = Medical Outcome Study Short Form 12; PCS = Physical Component Summary; MCS = Medical Component Summary.

1 = comparisons using chi-square tests and ANOVAs.

2 = comparisons using mixed effect logistic models (random effects = surgeon).

A significant effect of study site (p value = 0.003) was found.

Womac and physical quality of life yielded significant effects (both p values<0.01). “More optimistic” patients had a mean SF-12 physical component score of 29.4±1.1 while “more pessimistic” patients scored 34.6±1.3. A similar significant effect was found for Womac score, confirming that more disabled patients had higher expectations than their surgeons.

The other characteristic which differed between the two groups was professional category (p value = 0.02). Two thirds of patients who had “more pessimistic” expectations were from a liberal or senior officer professional category (62%), while two third of the “more optimistic” patients were employees or workers (66%).

## Discussion

To our knowledge, this is the first study to compare expectations of THA of patients and of their surgeons with a validated multi-dimensional expectations questionnaire.

Surgeons and patients expectations were both high as previously reported [23], reflecting the favourable outcomes usually observed after THA. Although it has been reported that surgeons could expect better outcomes than patients [18], we did not observe such a bias between surgeons' and patients' mean scores.

However, concordance was poor, as differences in expectations (surgeon's score – patients' score) showed high variability and poor correlation. Marked discrepancies could be seen in both directions, and were especially important for patients with lower expectations. Moreover, the methods of this study, which included solely patients for which both patients and surgeons had agreed for surgery, could have understated the discrepancy that really existed: if a patient has great expectations for surgery and the doctor does not, or vice versa, it is unlikely the operation will be scheduled. A study including all patients for which THA is discussed would thus be likely to find higher surgeon-patient discrepancies.

As shown on figure 1 and 2, the sample could be roughly divided into three groups explaining the peculiar structure of the Bland and Altman graph: one middle group of patients with high expectations and high surgeon-patient concordance, and two other groups, with patients who had “more optimistic” expectations than their surgeons on one side, and patients who had “more pessimistic” expectations on the other. Dividing the population into three tertiles-defined groups thus enabled us to compare patients in these two extreme groups.

The specific study of each item of the scale suggested that patients had higher expectations than their surgeons for the item “exercise and sports”. We also found that 12% of the sample wished to resume a sport which was not recommended after THA (table 1). It has been recently suggested that expectations regarding exercise and sports was one of the less frequently fulfilled [9]. Our study thus shows that this could be due to a mismatch in pre-operative expectations. This question of sports after THA might then be a cause of post-operative disappointment, and should be a matter of careful pre-operative discussion.

The study of factors affecting surgeons' and patients' expectations separately enabled us to highlight interesting differences in surgeons' and patients' point of views. Surgeons' ratings of expectations seemed to be significantly associated with hip-related clinical data (Womac, SF-12's physical component). On the other side, patients seemed to rate their expectations on criteria that were mainly psychological and non-hip-related: SF-12's mental component, trust in surgeon, and comorbidities. Patients with higher scores on trust in surgeon and higher scores on SF-12's mental component were likely to have higher expectations.

Age significantly affected both patients' and surgeons' expectations in the same direction, so that no effect of age on differences in expectations was seen. The evidence on the role of age on disability and quality of life after THA is conflicting, but a large number of studies show that post-THA improvement is comparable in older and younger patients [1]. In our study, both patients and surgeons seemed to agree on being more prudent on the estimated outcome of surgery for older patients, having thus somewhat more pessimistic views than the literature.

When studying surgeon-patient differences in total expectations scores, we found that several parameters were associated with disagreements. First, patients with low functional status and physical quality of life tended to be “more optimistic” than their surgeons. It has been shown that patients with poor functional status have high expectations for THA [6], and are at high risk of having unfulfilled expectations [9]. This study confirms that more disabled patients have higher expectations than surgeons before THA, which could mean unrealistic expectations, and that special attention should be given when informing these patients about their expected outcome.

Professional category significantly affected surgeon-patient agreement. Patients who were from a lower income category were often in the “more optimistic” group. The role of such socio-demographic variables on patients expectations has already been noted by other authors [5], [6], and should probably be also taken into account when delivering pre-operative information to patients.

We found significant effects of centres and surgeons on surgeons' ratings of expectations, which in turn influenced surgeon-patient differences in expectations. In contrast, neither patients' general characteristics nor patients' expectations were significantly different according to surgeons or centres. This is an interesting finding, as expectations of surgeons have not to our knowledge been described or compared by this kind of scale before, and further study on this subject could be of interest, as this effect might be related, at least partly, to the psychological profiles of each care providers. We chose not to standardize surgeons' evaluations, as the objective of this work was to study surgeon-patient concordance and communication for each surgeon–patient pair, rather than the appropriateness of patients' expectations in reference to a standard. It is unlikely that this surgeon effect on difference in expectations affected results of comparison tests between “more optimistic” and “more pessimistic” patients, as statistical analyses using mixed effect models and taking into account the clustered structure of the data, gave similar results.

Surprisingly, the three subscales evaluating surgeon-patient communication during clinical visits did not have any significant effect on surgeon-patient agreement in our study. A previous study noted that these scales, and especially information given by surgeons during clinical visit, predicted surgeon-patient concordance in expectations about THA [19]. An explanation could be that in our study, information was delivered not only during visits to surgeons, but also during structured information meetings with physiotherapists or by information leaflets and web sites. As our patients' interviews could take place at different times during this process, patients' assessments of provided information could vary.

This study has several strengths. The scarcity of missing data, due to the study protocol, which used telephone interviews to collect data, lowered the risks of bias. A unique evaluator performed all interviews, to ensure that questionnaires were understood and answered the same way by patients. Precautions were taken during our study to ensure that patients' and surgeons' assessments were done independently of each other.

As the aim of the study was to compare patients' and surgeons' judgments of the likely benefit from surgery, we chose to study “probability expectations”. We thus used a slightly modified question format, asking patients how much improvement they expected in each domain rather than how important each domain was for them. This modified question has been used before and validated for this questionnaire [5], [23]. The assessor ascertained during interviews that the question and its underlying concept were clearly understood by patients. The study of “value expectations” and relative importance of different domains for patients is a complementary approach in understanding patients' point of view and has been addressed by several authors [6], [31].

Several items of the questionnaire were often considered “not applicable” by patients, mostly because patients never found themselves in such situations. When calculating the total score, scoring zero for such items could have made total scores lower, even when patients had high expectations on all applicable items. We chose to calculate total scores by omitting items which were considered inapplicable by patients and dividing scores by the number of applicable items. The two different methods of scoring gave very different scores (data not shown), and this question should be raised when using this questionnaire.

This study has several limitations. Telephone interviews were performed at different times before surgery, and it has been shown that patients' expectations can change over time [32], although it is not clear how patients expectations are built and modified during the pre-surgical period. We believe that these variations were small in our study, as delay before surgery did not significantly affect surgeon-patient agreement.

Another limitation lies in the lack of calibration of measures between surgeons and patients before the study. Although the same evaluator delivered questionnaires to surgeons and assessed patients, it is not sure that surgeons and patients assessed their expectations in the same manner. However, the comparability of surgeons' and patients' scores on this specific scale was shown in a previous study [23], where patients' evaluations through telephone interviews were compared to a reference rating from surgeons. Comparison showed that 49% of patients had scores that were within 6 points of surgeons' values before, and 54% after a specific educative intervention.

Patients were classified as more optimistic or more pessimistic in comparison to their surgeons' expectations, which could be a matter of discussion, as surgeons' expectations do not seem to be a good predictor of patient satisfaction after total knee arthroplasty [33]. It is not known which of both assessments best predicts patient outcome and satisfaction after THA, and this will be a topic for further research.

In conclusion, surgeons and patients often did not agree on what to expect from THA, but there was no systematic bias between both evaluations. Patients had higher expectations than surgeons on post-operative sports. Patients rated their expectations according to trust in surgeon and mental quality of life, whereas surgeons considered disability and physical quality of life. Patients with higher disability expected higher outcome than their surgeons.
